# Racial and socioeconomic disparities in surgical management and outcomes in pancreatic adenocarcinoma: a single-center experience in the last 13 years

**DOI:** 10.1186/s12885-025-14588-w

**Published:** 2025-07-25

**Authors:** Dauris Rosario Lora, Sarah Herrera Mercedes, Zoe Post, Wojciech Blogowski

**Affiliations:** 1 Department of Internal Medicine, RUSH University Medical Center, Chicago, IL USA; 2 Division of Digestive Diseases and Nutrition, RUSH University Medical Center, 1725 W Harrison Str POB STE 207, IL 60612 Chicago, USA; 3 Institute of Medical Sciences, University of Zielona Gora, Zielona Gora, Poland

**Keywords:** African American, Asian, Healthcare disparities, Hispanic/Latino, Race, Socioeconomic level, Pancreatic Cancer, Surgery, Underrepresented populations

## Abstract

**Background:**

Pancreatic adenocarcinoma (PaC) is an aggressive cancer with a poor prognosis. While disparities in surgical management and outcomes have been reported, most studies use outdated or population-level data, limiting their relevance in modern clinical settings. This study examined the impact of race and socioeconomic status on pancreatic resection rates and survival outcomes in PaC patients.

**Methods:**

A retrospective analysis of 525 patients diagnosed with PaC at a single institution (2010–2024) was conducted. Demographics, tumor characteristics, resection rates, and survival outcomes were assessed. Socioeconomic status was inferred from zip codes. Logistic regression and Cox proportional hazards models were used to evaluate treatment access and survival.

**Results:**

African American patients had lower resection rates than White patients (20.0% vs. 36.1%; *p* < 0.001), even after adjusting for resectable stages. Resection likelihood was reduced by being African American (OR 0.27; *p* < 0.001), older age (OR 0.97/year; *p* = 0.007), advanced stage (OR 0.09; *p* < 0.001), and lower education (OR 0.86; *p* = 0.003). Mean survival was shorter for African Americans than White patients (405.7 vs. 426.8 days; *p* < 0.001) but nonsignificant after adjustments (HR 1.19; *p* = 0.34).

**Conclusions:**

Racial and socioeconomic disparities persist in PaC surgical management, impacting outcomes. Addressing these inequities through improved access to care is essential for achieving more equitable treatment.

## Introduction

Pancreatic adenocarcinoma (PaC) is an aggressive malignancy and the fourth leading cause of cancer-related mortality in the United States. The prognosis remains grim, with a 5-year survival rate of just 12.5%, according to recent data from the American Cancer Society. Approximately 80% of pancreatic cancer cases are identified at an advanced stage, often with metastasis to distant organs. Sadly, about 85% of PaC cases are considered unresectable at diagnosis. For the small percentage of cases where PaC is detected early enough to be resectable, surgery offers the best chance for cure and long-term survival. Over the past decade, 5-year survival rates following pancreatic resection have improved, ranging from 11 to 25%, a notable increase from prior decades when the survival rate was as low as 3% [1–3]. In a review article by Kolbeinsson et al., the median 5-year survival after surgical resection in modern reports is approximately 20% [4]. Other studies report post-resection survival rates between 22 and 34.5% [1, 5].

In recent years, disparities in pancreatic cancer outcomes have drawn increased attention. Previous research has identified African-American patients with PaC as a demographic facing significant challenges, including reduced access to specialist care, surgical resection, and chemotherapy, alongside worse survival outcomes [6]. Evidence suggests that Black patients with PaC are less likely to receive appropriate treatment [7]. A population-based study of 278,936 patients with pancreatic ductal adenocarcinoma (PaC) from the National Cancer Database found that white patients had longer median survival times compared to African-American patients. The study also revealed that African-American patients presented at younger ages, had more advanced disease at diagnosis, and were less likely to undergo surgical resection at early stages compared to white patients [8]. These disparities are thought to be multifactorial, though socioeconomic factors remain underexplored in this context.

While previous studies have extensively documented racial and socioeconomic disparities in pancreatic cancer treatment, most have relied on broad population-level data or national cancer registries, limiting their ability to capture contemporary trends in real-world clinical settings. Additionally, many of these studies predate modern therapies for PaC and the growing awareness of healthcare disparities, leaving the current impact of race and ethnicity on survival outcomes unclear. Our study builds on prior research by analyzing a recent, single-institution cohort over 13 years, providing a more detailed, patient-level assessment of these disparities. Specifically, we aim to determine whether socioeconomic factors, such as income and education, contribute to differences in both access to and timeliness of surgical management.

## Methods

### Ethics statement/declaration

This study was performed in accordance with appropriate regulations and guidelines highlighted in the “*World Medical Association Declaration of Helsinki – Ethical Principles for Medical Research Involving Human Subjects*”. The study protocol was approved by the Institutional Review Board (IRB) of the RUSH University (ORA #: 23111605-IRB01), and was compliant with all regulations established by the Health Insurance Portability and Accountability Act. In this study, informed consent was not obtained from individual participants, as it was a retrospective chart review of patients. The need for informed consent was waived by the aforementioned IRB under 45 CFR 164.512(i)(2)(ii).

### Data collection and participants

This retrospective, descriptive study examined adult patients diagnosed with pancreatic adenocarcinoma at Rush University Medical Center between January 2010 and January 2024. Working with the RUSH Department of Informatics, we extracted data from electronic healthcare medical records relating to initial encounters coded with ICD-10 code 157.1 for the diagnosis of “Malignant neoplasm of body of pancreas”. This data included patient demographics and the date the ICD code was added to their medical records. Inclusion criteria for the study encompassed patients aged 18 years or older with a confirmed diagnosis of pancreatic adenocarcinoma, established through both abdominal imaging and histopathologic evaluation. Patients were excluded if imaging or histopathologic documentation was absent from the medical record; if they were diagnosed with an alternative primary pancreatic or adjacent malignancy (e.g., neuroendocrine tumors, gallbladder cancer, or metastatic disease involving the pancreas); or if residential zip code data were unavailable, precluding linkage to socioeconomic information from the American Community Survey. Additionally, patients lacking sufficient documentation to verify clinical stage or treatment history were excluded from the analysis.

We reviewed patient electronic medical records to verify each diagnosis independently and directly record baseline demographic characteristics, such as age, sex, ethnicity, and zip code. The stage of pancreatic adenocarcinoma (PaC) was documented within one month of diagnosis using staging definitions from the American Joint Committee on Cancer (AJCC), and as defined in the patient’s initial visit. It is crucial to emphasize that treatment decisions, including the determination of surgical candidacy and plans for pancreatic resection, were made through a multidisciplinary tumor board at RUSH University Medical Center. These decisions were reached through a collaborative expert consensus, taking into account the clinical presentation, tumor characteristics, patient comorbidities, and individual patient preferences. The timing and history of pancreatic resections were also noted. Survival time was measured in days, starting from the initial ICD code placement date to the date of death or hospice enrollment, where available. Similarly, days to resection were measured as the total amount of days between the date of surgical resection if performed and the initial date of diagnosis. Additionally, patient zip codes at their first encounter were collected to estimate median income and educational level, using the most recent data from the American Census Survey Data from 2022. Specifically, educational attainment was estimated based on patients’ residential ZIP codes at the time of their initial pancreatic adenocarcinoma diagnosis. For each ZIP code, we extracted the proportions of residents who had completed a high school diploma or equivalent, a bachelor’s degree, or a graduate/professional degree. These proportions were incorporated as continuous variables to represent the educational profile of each patient’s community. Education level was reported as high school degree or equivalent, Bachelor’s degree, or Professional/Graduate Degree. Income was grouped into categories of Lower class (<$30,000), Lower-middle class ($30,001 - $58,020), Middle class ($58,021 - $94,000), Upper-middle class ($94,001 - $153,000) and Upper Class (>$153,000).

### Statistical analysis

Continuous variables are reported as means with standard deviations (SD), and categorical variables are presented as frequencies and percentages. Group comparisons were performed using crosstabulation. Univariate comparisons of continuous variables were conducted using the Student’s t-test, while one-way ANOVA was used to compare multiple means. Chi-square was utilized for the analysis of categorical variables. A multivariate logistic regression to look for predictors for resection was performed using a conditional approach where *p* < 0.0.5 was used as a cut-off for entry and *p* > 0.20 for removal. A sub-group analysis was done to look at differences in survival time between patients who underwent surgical resection. A Cox Regression was performed to look for disparities in survival time. Statistical analyses were conducted using SPSS 29.2 for Windows (SPSS Inc.). A two-sided P-value of < 0.05 was considered statistically significant for all tests.

## Results

### Patients’ characteristics

**Fig. 1:**
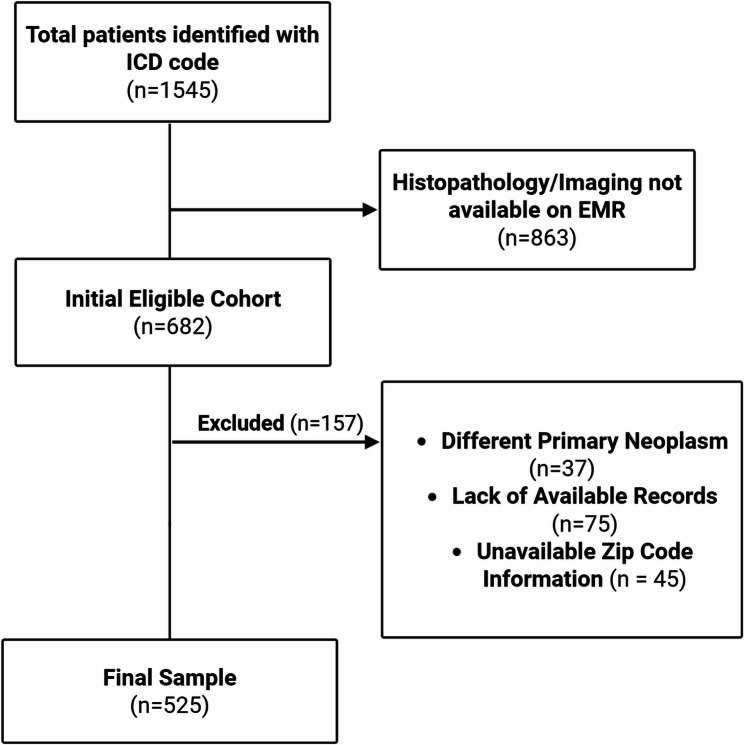
Cohort Selection Flow Diagram. Flow chart depicting the process of patient selection for the analytic cohort. A total of 1,545 patients were initially identified based on ICD-10 codes for pancreatic cancer between January 2010 and January 2024. Of these, 682 patients met preliminary inclusion criteria based on the availability of abdominal imaging and histopathologic confirmation. Following detailed chart review, 157 patients were excluded due to alternative primary malignancies (*n* = 37), incomplete or unavailable medical records (*n* = 75), or missing zip code data required for linkage to socioeconomic information (*n* = 45). The final analytic cohort included 525 patients

Between January 2010 and January 2024, a total of 1,545 patients were initially identified at our institution with a diagnosis of pancreatic cancer based on ICD-10 coding. Of these, 682 patients met preliminary inclusion criteria, defined by the availability of abdominal imaging and histopathologic confirmation, as determined through filters applied by our institutional informatics team. Following comprehensive chart review, 157 patients were excluded as presented on Fig. 1. The final analytic cohort comprised 525 patients. Of these patients, 295 (56.2%) were female, and 230 (43.8%) were male. 37.1% were African-American, while 43.2% were White. Hispanics were the following biggest population of 67 individuals (12.8%). The mean age at diagnosis was 66.1 years. The mean median income across the cohort was $78,361, with a relatively even distribution of patients across different income classes, except for the Upper-Class category, of which only 9 (1.7%) patients qualified. Based on zip code data, the average percentage of residents with a high school diploma or equivalent was 24.2%, while the average percentages for those with a Bachelor’s degree and a Graduate/Professional degree were 48.9% and 48.0%, respectively. These percentages represent mean values across zip codes and are not meant to sum to 100%, as each category was analyzed separately.

The most common location of pancreatic lesions identified on imaging was the pancreatic head, with 298 patients (56.8%) presenting with a detectable mass in this region. The mean lesion size was 3.46 cm. At diagnosis, nearly half of the patients (244, 46.5%) had a Stage IV diagnosis at their initial encounter. The median overall survival time for the cohort was 436.6 days. A total of 154 patients (29.3%) underwent pancreatic resection, with the mean time from diagnosis to surgery being 129.9 days. These patients were further evaluated in even greater detail, as specified below.

### Pancreatic resection and sociodemographic factors

Our analysis of pancreatic resection rates by ethnicity and race revealed that Black patients were significantly less likely to undergo resection compared to other racial groups (*P* < 0.001) (Table 1). Specifically, only 20.0% of African American patients in our cohort underwent resection, in contrast to 36.1% of White patients. Among all patients who received a resection, 53.2% were White, while only 25.3% were African American. Albeit not statistically significant, those who underwent resection were, on average, younger than those who did not. Notably, this disparity persisted despite similar diagnosis rates at Stages I and II (Table 2), where most resections occurred, across ethnicities in our cohort. Additionally, the educational level did not significantly influence the rates of pancreatic resection.

**Table 1:** Sociodemographic factors and rates of pancreatic resection. Reported as n (%), otherwise reported as mean and (SD)

	Pancreatic Resection		*p*-value
	Yes (*n* = 154)	No (*n* = 371)	
Age at diagnosis (y)	64.7 (11.4)	66.7 (10.8)	0.054
Body Mass Index (kg/m2)	26.8 (6.8)	27.1 (5.9)	0.600
Sex			
Male	74 (32.2%)	156 (67.8%)	0.207
Female	80 (27.1%)	215 (72.9%)	
Race/Ethnicity			
Hispanic	27 (40.3%)	40 (59.7%)	< 0.001
African-American	39 (20.0%)	156 (80.0%)	
White/Caucasian	82 (36.1%)	145 (63.9%)	
Asian	4 (36.4%)	7 (63.6%)	
Other	2 (8.0%)	23 (92.0%)	
AJCC Stage at Diagnosis			
I	52 (60.5%)	35 (39.5%)	< 0.001
II	48 (41.4%)	68 (58.6%)	
III	24 (44.4%)	30 (55.6%)	
IV	29 (11.9%)	215 (88.1%)	
Unstaged	1 (4.2%)	23 (95.8%)	
Size of Lesion (cm)	2.98 (1.54)	3.64 (1.52)	< 0.001
Location of Lesion			
Head	101 (33.9%)	197 (66.1%)	0.047
Body	16 (20.0%)	64 (80.0%)	
Tail	11 (19.3%)	46 (80.7%)	
Multiple Portions	14 (22.6%)	48 (77.4%)	
Other	5 (35.7%)	9 (64.3%)	
Median Income (US$)	81,258.6 (30,860.1)	77,158.9 (33,538.7)	< 0.001
Class Per Income			
Lower-middle class	43 (24.3%)	134 (75.7%)	0.297
Middle class	58 (32.2%)	122 (67.8%)	
Upper-middle class	51 (32.1%)	108 (67.9%)	
Upper class	2 (22.2%)	7 (77.8%)	

**Table 2:** Race/Ethnicity vs stage of PaC at diagnosis

Stage of PaC	Hispanic (*n* = 67)	Black/African-American (*n* = 195)	White/Caucasian (*n* = 227)	Asian (*n* = 11)	Other (*n* = 25)
Stage I	15 (22.4%)	23 (12.3%)	43 (18.9%)	1 (9.1%)	4 (16.0%)
Stage II	16 (23.9%)	46 (23.6%)	48 (21.1%)	4 (36.4%)	2 (8.0%)
Stage III	8 (11.9%)	20 (9.7%)	19 (8.4%)	2 (18.2%)	5 (20.0%)
Stage IV	25 (37.3%)	93 (47.7%)	110 (48.5%)	4 (36.4%)	12 (48.0%)
Not Staged	3 (4.5%)	12 (6.2%)	7 (3.1%)	0 (0.0%)	2 (8.0%)

A subgroup analysis including only Stage I and II patients demonstrated similar results (Table 3). Among the 100 patients who underwent pancreatic resection, the mean age at diagnosis was 65.27 years (SD: 10.65), compared to 68.21 years (SD: 11.24) for the 103 patients who did not undergo resection (*p* < 0.001). In terms of race/ethnicity, Caucasians had the higher percentage of resection within their group (58.2%), while African-Americans had the lowest rate (38.6%). The racial distribution demonstrated a statistically significant association with resection status (*p* = 0.011). Median household income was also significantly higher among the resected group, with a mean income of $81,399.53 (SD: $31,616.92) compared to $78,671.75 (SD: $33,472.84) in the non-resected group (*p* < 0.001).

**Table 3:** Sociodemographic factors and rates of pancreatic resection in stage I/II patients. Reported as n (%), otherwise reported as mean and (SD)

	Pancreatic Resection		*p*-value
	Yes (*n* = 100)	No (*n* = 103)	
Age at diagnosis (y)	65.27 (10.65)	68.21 (11.24)	< 0.001
Body Mass Index (kg/m2)	27.55 (7.05)	26.59 (5.81)	< 0.001
Sex			
Male	24 (42.9%)	32 (57.1%)	0.755
Female	24 (40.0%)	36 (60.0%)	
Race/Ethnicity			
Hispanic	18 (58.1%)	13 (41.9%)	< 0.011
African-American	27 (38.6%)	43 (61.4%)	
White/Caucasian	53 (58.2%)	38 (41.8%)	
Asian	2 (40.0%)	3 (60.0%)	
Other	0(0.0%)	6 (100.0%)	
Size of Lesion (cm)	2.78 (1.16)	3.05 (1.09)	< 0.001
Location of Lesion			
Head	73 (50.0%)	73 (50.0%)	0.673
Body	8 (34.8%)	15 (65.2%)	
Tail	7 (50.0%)	7 (50.0%)	
Multiple Portions	7 (58.3%)	5 (41.7%)	
Other	3 (50.0%)	3 (50.0%)	
Median Income (US$)	81,399.53 (31,616.92)	78,671.75 (33,472.84)	< 0.001
Class Per Income			
Lower-middle class	46 (46.8%)	55 (53.2%)	0.938
Middle class	48 (48.7%)	53 (51.3%)	
Upper-middle class	52 (52.5%)	47 (47.5%)	
Upper class	50 (50.0%)	50 (50.0%)	

When examining pancreatic resection rates by median income and income class, we found that Middle and Upper-middle Class patients were more likely to undergo resection, representing 37.7% and 33.1% of cases, respectively. However, this did not reach statistical significance. Those patients who underwent pancreatic resection had a significantly higher median income than those who did not ($81,259.6 vs. $77,158.9 *p* < 0.001). Additionally, when assessing median income by ethnicity, African Americans had the lowest median income across all racial groups ($59,612.0, *p* < 0.001). They represented the 66.1% of patients in our cohort falling into the Lower-middle Class category based on income (*p* < 0.001) (see Table 4). A subset analysis of patients diagnosed with Stage I/II PaC demonstrated similar statistically significant results, indicating that the previously mentioned trends also apply to patients potentially resectable at the time of diagnosis.

**Table 4:** Mean income as well as class distribution across ethnicities. Reported as n (%), otherwise reported as mean and (SD)

Variable	Hispanic (*n* = 67)	Black/African-American (*n* = 195)	White/Caucasian (*n* = 227)	Asian (*n* = 11)	Other (*n* = 25)	Total (*n* = 525)	*p*-value
Mean Income ($)	66,717.3 (23,387.6)	59,612.0 (25,458.4)	97,670.9 (30,938.5)	87,491.4 (24,553.0)	76,473.2 (26,030.3)	78,361.8 (32,799.8)	< 0.001
Lower-middle Class	32 (18.1%)	117 (66.1%)	22 (12.4%)	1 (0.6%)	5 (2.8%)	177	< 0.001
Middle Class	23 (12.8%)	55 (30.6%)	81 (45.0%)	6 (3.3%)	15 (8.3%)	180	
Upper-middle Class	12 (7.5%)	23 (14.5%)	115 (72.3%)	4 (2.5%)	5 (3.1%)	159	
Upper Class	0 (0.00%)	0 (0.00%)	9 (100.0%)	0 (0.00%)	0 (0.00%)	9	

The time from diagnosis to resection varied significantly across racial and income groups. Notably, African American patients who underwent resection had the shortest mean interval between diagnosis and surgery, with a mean of 101.5 days (SD 98.0; *p* < 0.001), compared to other racial groups. This was followed by Hispanic patients, with a mean time of 138.9 days (SD 137.7), and White/Caucasian patients, who had a mean of 139.5 days (SD 136.8).

In contrast, when stratified by income, patients from the Middle Class experienced the longest mean time to resection, at 151.5 days (SD 142.3; *p* < 0.001). Upper-Middle Class patients had a mean interval of 120.5 days (SD 114.5), while Lower-Middle Class patients had a mean of 117.7 days (SD 114.7).

A multivariate logistic regression analysis (Table 5) identified several significant predictors of undergoing pancreatic resection in patients with pancreatic cancer. Younger age was associated with an increased likelihood of resection, with an adjusted odds ratio (AOR) of 0.97 (95% CI 0.95–0.99, *p* = 0.007). Racial disparities were observed, as African-American/Black patients had significantly lower odds of undergoing resection compared to White patients (AOR 0.27, 95% CI 0.14–0.53, *p* < 0.001). Socioeconomic factors such as education level played a role, with high school graduates having a reduced likelihood of resection (AOR 0.86, 95% CI 0.78–0.95, *p* = 0.003) compared to those with higher educational attainment. As expected, tumor-related factors, particularly the stage of cancer, were also significant; patients with Stage II, III, and IV cancers were progressively less likely to undergo resection compared to those with Stage I disease, with Stage IV patients having the lowest odds (AOR 0.09, 95% CI 0.04–0.17, *p* < 0.001).

**Table 5:** Binary logistic regression for pancreatic resection

Characteristic	AOR	*p*-value	95% CI for AOR
Age at Diagnosis	0.97	0.007	[0.95–0.99]
Race			
White	Referent		
African-American/Black	0.27	< 0.001	[0.14–0.53]
Hispanic	0.76	0.461	[0.37–1.58]
Asian	0.5	0.366	[0.11–2.25]
Other	0.09	0.004	[0.02–0.47]
Median Income	1	0.918	[1.00–1.00]
Social Class			
Lower-middle Class	Referent		
Middle Class	1.02	0.966	[0.43–2.42]
Upper-middle Class	1.52	0.599	[0.32–7.18]
Upper Class	0.84	0.918	[0.03–24.42]
Education Level			
High School Graduate	0.86	0.003	[0.78–0.95]
Bachelor’s Degree	0.9	0.002	[0.84–0.96]
Graduate or Professional Degree	0.96	0.283	[0.90–1.03]
Location of Lesion			
Head	Referent		
Body	0.67	0.276	[0.33–1.37]
Tail	0.79	0.597	[0.33–1.90]
Multiple Portions	0.89	0.756	[0.39–1.97]
Size of Lesion	0.84	0.076	[0.70–1.02]
Stage at Diagnosis			
Stage I	Referent		
Stage II	0.41	0.007	[0.22–0.79]
Stage III	0.61	0.221	[0.28–1.35]
Stage IV	0.09	< 0.001	[0.04–0.17]
Unstaged	0.04	0.003	[0.004–0.33]

### Analysis of the survival time

Overall survival time was significantly longer for patients diagnosed at earlier stages of pancreatic cancer (PaC). Significant differences in mean survival times were observed across racial/ethnic groups at Stage II (*p* = 0.045). At this stage, Hispanic patients had the longest mean survival time (1,011.0 days, SD 433.1), while patients categorized as “Other” had the shortest (174.5 days, SD 66.5). No statistically significant survival differences across racial/ethnic groups were observed for patients diagnosed at Stage I (*p* = 0.162) Stage III (*p* = 0.990), or Stage IV (*p* = 0.352). Significant differences in mean survival times were also observed across ethnicities and income levels. African American patients demonstrated a significantly shorter survival time compared to Caucasian patients (405.7 vs. 426.8 days, *P* < 0.001). Interestingly, patients from the upper-middle class exhibited lower mean survival times (389.9 days, *P* < 0.001) compared to other social classes (Table 6).

**Table 6:** Mean survival times across cohorts

Variable	Mean Survival Time	SD	*p*-value
Pancreatic Resection			< 0.001
No (*n* = 185)	359.9	413.8	
Yes (*n* = 68)	645.4	522.9	
Stage of PaC			< 0.001
Stage I (*n* = 37)	673.0	594.7	
Stage II (*n* = 57)	537.6	569.7	
Stage III (*n* = 23)	479.2	325.4	
Stage IV (*n* = 127)	316.2	331.1	
Not Staged (*n* = 9)	415	534.1	
Race/Ethnicity			< 0.001
Hispanic (*n* = 40)	546.0	694.5	
Black/African-American (*n* = 94)	405.7	420.6	
White/Caucasian (*n* = 103)	426.8	379.3	
Asian (*n* = 4)	792.1	791.5	
Other (*n* = 12)	279.9	248.0	
Class Per Income			0.297
Lower-middle Class (*n* = 90)	409.7	375.3	
Middle Class (*n* = 93)	495.3	563.1	
Upper-middle Class (*n* = 67)	389.9	418.9	
Upper Class (*n* = 3)	468.0	157.8	

A subset analysis of patients who underwent surgical resection revealed that African American patients experienced notably longer survival times (810.8 days, SD 533.9, *P* < 0.001) compared to other racial groups, except Asians (1039.7 days, SD 756.4, *n* = 3). White/Caucasian and Hispanic patients had mean survival times of 515.6 days (SD 435.5) and 670.5 days (SD 634.8), respectively. Regarding income class, patients from the middle class had poorer survival outcomes (602.9 days, SD 518.4, *P* < 0.001) compared to other classes, followed by those from the upper-middle class (670.9 days, SD 619.5) and lower-middle class (692.0 days, SD 472.0).

In the Cox regression analysis of survival time (Table 7) several factors were evaluated for their impact on patient outcomes. Age at diagnosis showed a statistically significant hazard ratio (HR) of 1.014 (95% CI: 1.000-1.029, *p* = 0.047), suggesting that each additional year at diagnosis slightly increased the risk of mortality. In the racial subgroup analysis, African-American/Black patients had an HR of 1.19 (95% CI: 0.84–1.70, *p* = 0.336), and Hispanic patients had an HR of 0.86 (95% CI: 0.55–1.34, *p* = 0.501), compared to the referent group, White/Caucasian, though these results were not statistically significant. Asian patients demonstrated an HR of 2.08 (95% CI: 0.62–6.98, *p* = 0.236). Socioeconomic status showed some effect, particularly with lower-middle-class individuals being the referent; however, none of the other social classes demonstrated statistically significant differences in risk. Regarding stage at diagnosis, patients with Stage IV disease had a significantly increased risk of mortality compared to Stage I (HR 2.88, 95% CI: 1.78–4.66, *p* = 0.001), indicating a marked worsening prognosis with advanced disease. Other evaluated factors, such as sex, lesion size, education level, and location of the lesion, did not show significant associations with survival in this cohort.

**Table 7:** Results of Cox regression analysis

Characteristic	Hazard Ratio (HZ)	*P*-value	95% CI for HZ
Female Sex	1.118	0.448	[0.837–1.494]
Age at Diagnosis	1.014	0.047	[1.000–1.029]
Race			
White/Caucasian	Referent	0.053	
African-American/Black	1.19	0.336	[0.835–1.697]
Hispanic	0.858	0.501	[0.549–1.340]
Asian	2.079	0.236	[0.619–6.981]
Other	2.461	0.012	[1.218–4.973]
Median Income	1	0.089	[1.000–1.000]
Social Class			
Lower-middle Class	Referent	0.035	
Middle Class	0.92	0.755	[0.544–1.555]
Upper-middle Class	2.135	0.134	[0.793–5.753]
Upper Class	2.563	0.391	[0.298–22.057]
Education Level			
High School Graduate	0.992	0.791	[0.936–1.052]
Bachelor’s Degree	1.011	0.651	[0.965–1.058]
Graduate or Professional Degree	0.989	0.533	[0.953–1.025]
Size of Lesion	1.039	0.32	[0.963–1.121]
Location of Lesion			
Head		0.322	
Body	0.877	0.498	[0.601–1.281]
Tail	1.324	0.286	[0.791–2.216]
Multiple Portions	0.675	0.111	[0.416–1.094]
Other	1.129	0.797	[0.449–2.840]
Unavailable	2.435	0.39	[0.320–18.530]
Stage at Diagnosis			
Stage I	Referent	0.001	
Stage II	1.513	0.085	[0.944–2.424]
Stage III	1.521	0.169	[0.837–2.766]
Stage IV	2.875	0.001	[1.775–4.655]
Unstaged	2.33	0.068	[0.940–5.773]

### Geographic access and outcomes

Among the 525 patients included in our analysis, only 4 (0.8%) resided in rural ZIP codes as defined by housing density, while the remaining 521 (99.2%) were classified as urban. The average straight-line distance from patients’ home ZIP code to our institution was 13.7 miles (SD 15.8), ranging from 0 to 147.7 miles. Distance to the institution differed significantly by race/ethnicity (*p* < 0.001). White patients lived the farthest from our center (mean 19.1 miles), followed by patients categorized as Other (17.4 miles), while Hispanic and African-American patients lived closer (means 8.4 and 9.0 miles, respectively).

Stage at diagnosis was also associated with geographic distance (*p* = 0.021). Patients diagnosed at stage I lived significantly farther from the institution (mean 18.8 miles) compared to those diagnosed at later stages (means 11.1–13.3 miles). Bonferroni post-hoc analysis confirmed significant differences between stage I and stages III and IV.

Patients who underwent pancreatic resection had a greater mean distance to the hospital (16.6 miles vs. 12.5 miles, *p* = 0.008). Among patients with early-stage disease (stage I/II only), those who underwent surgery lived significantly farther from the institution (mean 19.4 miles vs. 12.0 miles; *p* < 0.001).

There was no significant correlation between geographic distance and overall survival (Pearson *r* = − 0.063, *p* = 0.316).

## Discussion

The results of our study highlight significant racial and socioeconomic disparities in the surgical management and outcomes of pancreatic adenocarcinoma (PaC), consistent with existing literature while offering new insights into the persistence and underlying factors of these inequities. Our findings reveal that African American patients seem to be the most affected by these disparities, with overall significantly less likelihood to undergo pancreatic resection compared to their White counterparts, even after controlling for disease stage and other tumor-related factors.

Our findings are consistent with previous literature on racial and ethnic disparities in the treatment of pancreatic cancer [6, 8–10]. Heller et al. recently reported that African American patients with PaC were less likely to undergo surgical resection, even when presenting at earlier stages [8]. Similarly, Murphy et al. found that despite presenting with resectable disease and being recommended surgery at rates comparable to White patients, African American patients underwent significantly fewer resections, suggesting an underutilization of pancreatic surgery in Black patients [6]. Population studies using data from the Surveillance, Epidemiology, and End Results (SEER) database from 1992 to 2011 showed that minority groups, including African Americans, were less likely to receive treatments such as chemotherapy, radiation, or surgery, despite adjusting for various sociodemographic factors such as geographic location, socioeconomic status, stage, tumor location, and grade [9]. Singal et al. also reported similar findings, demonstrating lower resection rates among African Americans even after controlling for possible confounders [10]. 

A complex interplay of multiple factors is likely the driver of the disparities in pancreatic cancer treatment among ethnicities. Provider-level influences have been identified as contributing to these differences, with previous studies reporting lower rates of surgical resection recommendations for African American patients. These disparities may be partially attributed to physician biases or the unequal dissemination of information regarding treatment options [11–13]. Furthermore, African American patients are less likely to consult with an oncologist compared to other racial groups, which may further limit access to appropriate care [14]. On the patient side, data suggest that African American patients are more likely to refuse pancreatic cancer-directed surgery compared to their White counterparts [11, 15, 16]. This reluctance may be rooted in a long-standing mistrust of the healthcare system within the African American community. This distrust is often amplified in interactions with White physicians, as studies have demonstrated that African American patients report greater trust and improved communication when treated by African American physicians; however, African American physicians make up a small percentage of the overall physician workforce, which limits opportunities for such culturally concordant care [17, 18]. Our analysis highlights that although such negative tendencies were reported and raised at the beginning of the 2000s’, they persist and apply to patients treated during the last decade.

In our study, socioeconomic factors were significantly associated with treatment access. Patients who underwent pancreatic resection had a significantly higher median income than to those who did not. Among all racial groups, African American patients had the lowest median income of approximately $60,000. Previous studies have shown that higher income is significantly associated with being offered or receiving treatment for pancreatic cancer, including surgery, regardless of the setting. These studies also found that African American patients had significantly lower average incomes [15, 19, 20]. Interestingly, unlike previous studies, we did not observe a significant relationship between educational level and undergoing pancreatic resection, which may be attributed to our cohort’s limited geographic scope and relatively smaller sample size.

It has been previously noted that African Americans often face delays in receiving treatment, including surgery, for pancreatic cancer [19, 21]. Chang et al. reported similar surgical resection rates across ethnicities and found no significant differences in the time from diagnosis to surgery among racial groups [22]. In contrast, our study identified disparities in time to surgery, with African American patients who underwent resection experiencing the shortest mean interval from diagnosis to surgery compared to other racial groups. Although limited data exist on treatment delays specific to surgical resection in pancreatic cancer, our findings suggest that, in our cohort, efforts were made to expedite surgical treatment for African American patients once they accessed surgical care. This may reflect an increased awareness of historical disparities in care and the need for rapid intervention due to historically worse outcomes in this population. It could also be influenced by the diverse healthcare workforce at Rush University, where representation plays a vital role in driving equitable and compassionate care. Moreover, differences in insurance coverage and the time required for therapy approval may have contributed to these findings.

Our logistic regression analysis provided further insight into the disparities in pancreatic cancer treatment. African American patients had significantly lower odds of undergoing surgical resection compared to White patients. This finding is consistent with previous multivariate analyses showing that being African American not only reduces the likelihood of receiving surgery but may also decrease the odds of receiving any form of treatment [14, 19, 20, 23]. Age was also a significant predictor, with younger patients more likely to undergo surgery. This aligns with existing literature, which has indicated that older patients are less likely to receive surgical interventions, likely due to concerns about frailty and poorer postoperative outcomes in the elderly population [15, 20, 24]. Socioeconomic factors, particularly education level, also showed a significant association with the likelihood of undergoing surgery. Patients with only a high school diploma had reduced odds of receiving resection. This finding reflects other studies that suggest lower educational attainment, while not necessarily a barrier to being recommended for surgery, may influence a patient’s decision to refuse or delay treatment, potentially due to limited understanding of their treatment options [15, 19]. Finally, as expected, those with early disease stages were more likely to undergo resection, than those with more advanced disease. This result mirrors existing literature that underscores the importance of early diagnosis in improving resection eligibility and survival outcomes. It also highlights the concerning trend that African American patients are more likely to present with advanced-stage disease (Stage II or IV), which further diminishes their chances of receiving curative treatment [8, 19]. 

Our unadjusted survival time analysis showed shorter mean survival times for African American patients, aligning with existing literature [8]. Notably, African American patients who underwent pancreatic surgery experienced a marked improvement in mean survival time, increasing from 405.7 days to 810.8 days, compared to a smaller improvement for White patients, whose mean survival time increased from 426.8 days to 515.6 days post-surgery. However, in our Cox regression analysis, after adjusting for both sociodemographic factors (Age, Sex, Race) and clinical characteristics (Stage at diagnosis, location and size of lesion), the difference in survival time between ethnicities became nonsignificant. The literature offers conflicting results on the impact of race on survival outcomes. Some studies have shown a persistent increase in mortality for African American patients even after adjusting for confounding factors [6, 8]. Compared to the larger population-based studies, the lack of statistical power in our study, might account for the inability to detect a significant effect.

Furthermore, we observed that geographic distance to our institution varied significantly by race and ethnicity, with White patients residing farther on average than Hispanic and African-American patients. Additionally, patients who underwent surgical resection and those diagnosed at earlier stages lived at greater distances from our center. These patterns suggest that individuals referred from more distant locations - potentially from community-based or suburban healthcare facilities - may have undergone earlier diagnostic workups or received expedited referrals to tertiary care.

Importantly, geographic distance was not significantly associated with overall survival, indicating that once patients are engaged in specialized care, proximity to the treatment center may not independently influence long-term outcomes. This finding aligns with prior studies reporting no adverse effect of travel distance on survival following pancreatic resection when care is delivered at high-volume academic institutions such as ours [25].

Our study provides an updated perspective on disparities in surgical management and outcomes in a well-characterized, diverse, and generalizable population, allowing for more detailed insights into the persistence of racial and socioeconomic inequities. We utilized direct data extraction from electronic medical records from a single healthcare system, ensuring accuracy and granularity in patient demographics, treatment timelines, and outcomes compared to studies relying on population databases. Additionally, logistic regression and Cox proportional hazards models allowed for a robust analysis of factors influencing both resection rates and survival, offering a comprehensive assessment of disparities in treatment access and outcomes. By controlling for key variables such as disease stage, income, and education, this study was able to isolate the effect of race on access to surgery, adding nuance to existing literature.

However, this study has also limitations. As a single-center study, the generalizability of our findings is constrained. While our cohort represents a relatively recent and sizable group of patients, the results may not fully apply to other healthcare settings, particularly those in the community or rural areas where healthcare disparities might be more pronounced. In our study we needed to exclude a population of patients due to incomplete documentation or ZIP code linkage failure, which affected individuals residing in rural or socioeconomically diverse areas. Compared to the included cohort, excluded patients tended to live in areas with lower educational attainment and showed a higher proportion of missing race/ethnicity data. Although these exclusions were necessary for consistency in data analysis, they may introduce some degree of selection bias and limit the generalizability of our findings to populations with more robust health record infrastructure or those residing in socioeconomically advantaged urban areas. Nonetheless, it is worth noting that previous research has demonstrated that patients from racial and ethnic minority groups often prefer providers who share their racial or gender identity, which can enhance trust and improve health outcomes. Given that the Chicago area – where this study was conducted – is among the most racially and ethnically diverse regions in the United States, with a similarly diverse healthcare workforce, our findings may still be generalizable to other urban settings with comparable demographic and provider diversity. Additionally, the retrospective design restricts our ability to establish causation between socioeconomic factors and treatment outcomes and account for patient preferences regarding surgery, which differ given cultural differences between ethnicities. Income and education were inferred from zip code data, which may not reflect individual-level variations accurately, and the influence of factors such as patient preference and provider recommendation, which could also contribute to disparities, was not explored in depth. Furthermore, another limitation of our analysis is the absence of access to outside hospital records. Many patients referred to our institution originate from facilities that do not share an integrated electronic health record system with ours. Consequently, we were unable to consistently capture information regarding the timing of referrals or any potential delays in care prior to presentation at our center. Finally, it is important to note that our study did not capture immigration status or distinguish between U.S.-born and foreign-born African American patients, as there is existing literature indicating potential cultural differences and distinct healthcare-seeking behaviors among U.S.-born versus foreign-born Black populations [26].

In summary, our study highlights persistent racial and socioeconomic disparities in the surgical management of pancreatic adenocarcinoma, which have a profound impact on patient outcomes. African American patients are significantly less likely to undergo surgical resection—the only potential curative treatment for this aggressive malignancy. These disparities persist even after adjusting for clinical factors, underscoring the ongoing influence of social determinants of health on access to care and survival. Our findings stress the critical need for targeted interventions to promote equitable access to surgical care and improve outcomes for African American patients. Strategies to enhance access to specialized care, increase patient education, and reduce provider bias are essential to narrowing these disparities. Additionally, future research focusing on intrinsic aspects of race, such as cultural nuances and differences, could offer further insight into the underlying causes of these disparities and inform more effective interventions.

## Data Availability

All data generated or analyzed during this study are included in this published article.
